# Anesthetic management in an adult moyamoya disease patient undergoing mitral valve plasty for severe mitral regurgitation

**DOI:** 10.1186/s40981-016-0039-4

**Published:** 2016-07-07

**Authors:** Kazutomo Saito, Hiroaki Toyama, Yutaka Ejima, Masanori Yamauchi

**Affiliations:** 1 Department of Anesthesiology, Tohoku University Hospital, 1-1 Seiryomachi, Aoba-ku, Sendai, 980-8574 Japan; 2 Division of Surgical Center and Supply, Sterillization, Tohoku University Hospital, 1-1 Seiryomachi, Aoba-ku, Sendai, 980-8574 Japan; 3 Anesthesiology and Perioperative Medicine, Tohoku University School of Medicine, 2-1 Seiryomachi, Aoba-ku, Sendai, 980-8575 Japan

**Keywords:** Moyamoya disease, Cardiopulmonary bypass, Intra-aortic balloon pumping, Sevoflurane, Preconditioning

## Abstract

**Background:**

Despite several previous reports, there are no established procedures for intraoperative management in moyamoya disease patients requiring cardiac surgery.

**Case presentation:**

Herein, we report the case of a 42-year-old man who was scheduled to undergo mitral valve plasty for severe mitral regurgitation. He had been diagnosed with moyamoya disease on the onset of cerebral ischemia at 41 years of age. During the cardiac surgical procedure, the patient was maintained on inhalation anesthesia with 1 to 1.5 % sevoflurane. Sevoflurane causes cerebral vasodilation followed by increased cerebral blood flow, and moreover we expected a sevoflurane preconditioning-induced neuroprotective effect. In addition, we used pulsatile perfusion support to maintain cerebral circulation with intra-aortic balloon pumping during the cardiopulmonary bypass. We aimed to keep the mean arterial pressure constantly above 70 mmHg. We were able to maintain regional cerebral oxygen saturation at 80 % of the baseline value, and could not detect the progression of neurological deficits using follow-up brain single photon emission computed tomography. The patient was discharged 16 days after admission.

**Conclusions:**

The details of the clinical course of his case will add to our knowledge regarding intraoperative management options in moyamoya disease patients requiring cardiac surgery. We suggest that pulsatile blood flow supported by intra-aortic balloon pumping and sevoflurane anesthesia for increasing cerebral blood flow and for possible neuroprotection may be efficacious for anesthetic management of moyamoya disease patients.

## Background

Moyamoya disease (MMD) is a chronic cerebrovascular disorder characterized by steno-occlusive changes of the terminal portion of the internal carotid arteries and the development of a network of abnormal collateral vessels [1]. In MMD patients with severe cerebrovascular disorders, prevention of cerebral ischemia or cerebral hemorrhage during cardiac surgery involving cardiopulmonary bypass (CPB) is extremely difficult because autoregulation of cerebral blood flow is impaired. Several previous reports have described intraoperative management in MMD patients requiring cardiac surgery [2–4], but there is no established evidence regarding anesthetic agents for maintenance of general anesthesia, effects of pulsatile perfusion, appropriate cerebral perfusion pressure and arterial carbon dioxide partial pressure (PaCO_2_) during CPB.

Herein, we report the successful management of a patient who had right hemiplegia due to MMD, and who underwent mitral valve plasty for severe mitral regurgitation (MR) without deterioration of neurological function.

## Case presentation

A 41-year-old man (height 171.5 cm, body weight 67 kg) was transferred to the regional medical center due to right hemiplegia and aphagia. Cerebral magnetic resonance imaging revealed cerebral infarction caused by occlusion of the left middle cerebral artery, while cerebral magnetic resonance angiography showed the development of a network of abnormal collateral vessels. Hence, the patient was diagnosed with MMD.

Before cerebral revascularization surgery, severe MR (III/IV) due to the prolapse of the P2 leaflet in the mitral valve was indicated by transthoracic echocardiography. Cardiac catheterization indicated elevated pulmonary arterial pressure (PAP) (systolic/diastolic/mean: 86/33/60 mmHg) and pulmonary capillary wedge pressure (32 mmHg) at the systemic arterial pressure of 120/83/102 mmHg. Hence, the patient was admitted to our university center for the surgical treatment of MR.

**Fig. 1:**
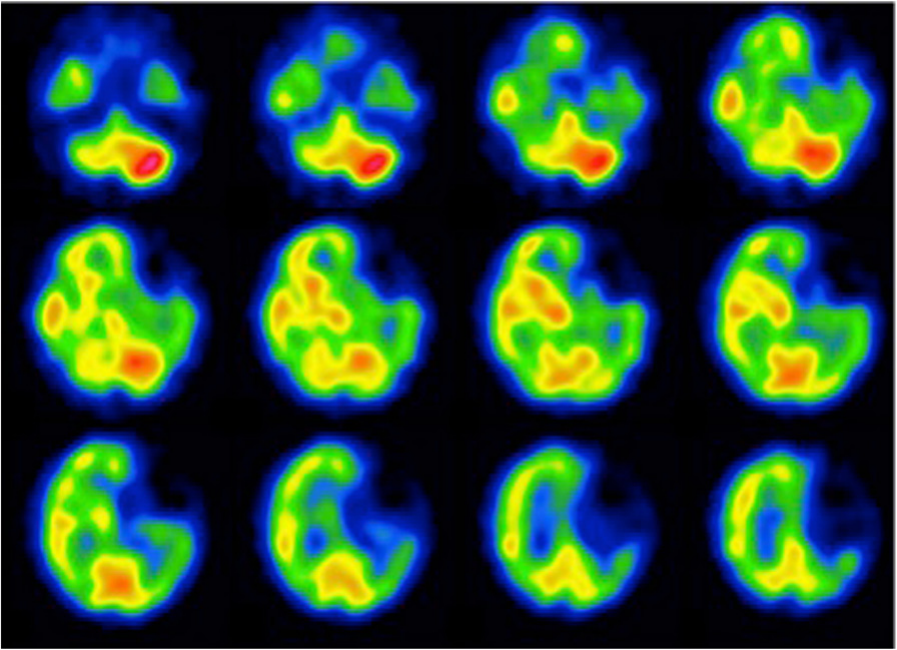
Preoperative single-photon emission computed tomography scan. A preoperative single-photon emission computed tomography scan taken at the regional medical center revealed reduced cerebral blood flow in the left cerebral hemisphere (especially in the external left frontal cortex)

Preoperative single-photon emission computed tomography revealed reduced cerebral blood flow in the left cerebral hemisphere (especially in the external left frontal cortex; Fig. 1). Neurosurgeons at our center judged that the patient did not have an indication for cerebral revascularization surgery, which is used to prevent ischemic complications during the perioperative period of mitral valve plasty. This was because his left frontal lobe showed extensive cerebral infarction and no cerebral infarction symptoms were observed in the right cerebral hemisphere. Therefore, mitral valve plasty without cerebral revascularization was chosen.

In the operation room, the patient’s monitoring of electrocardiogram, oxygen saturation, systemic arterial pressure via right radial artery catheter, bispectral index, and regional cerebral oxygen saturation (rSO_2_) at the right and left forehead (INVOS™ 5100C, Somanetics, USA) was initiated before the administration of general anesthesia. The rSO_2_ values for the left and right forehead were 72 and 81 %, respectively.

General anesthesia was induced by intravenous administration of 3 mg of midazolam, 0.4 mg of fentanyl, and 50 mg of rocuronium. After tracheal intubation, a transesophageal echocardiography (TEE) probe was inserted. Then, a central venous catheter and right heart catheter were inserted via the right internal jugular vein, and central venous pressure, PAP, cardiac output, and mixed venous oxygen saturation were measured. The nasopharyngeal temperature and urinary bladder temperature were also measured.

**Fig. 2:**
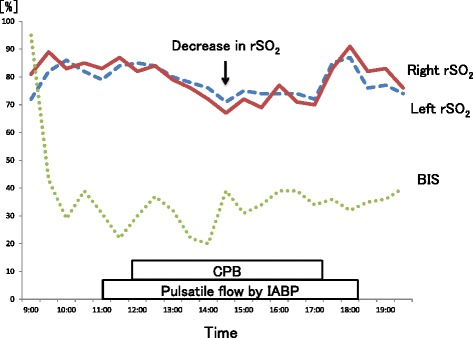
Intraoperative regional cerebral oxygen saturation measured by INVOS® and bispectral index. rSO_2_, regional cerebral oxygen saturation; BIS, bispectral index; CPB, cardiopulmonary bypass; IABP, intra-aortic balloon pumping

Before CPB, general anesthesia was maintained by inhalation of sevoflurane (1–1.5 % of end-tidal concentration). The patient’s PaCO_2_ was maintained between 38 and 42 mmHg. Intra-aortic balloon pumping (IABP) was placed at the start of surgery and the augmented pressure was maintained. The mean arterial pressure was constantly above 70 mmHg. Before CPB, rSO_2_ values were almost above 80 % on both sides (Fig. 2).

During CPB, administration of sevoflurane via the oxygenator was also continued because of its cerebrovascular dilatation activity and potential preconditioning effect against focal cerebral ischemia. PaCO_2_ was maintained between 45 and 50 mmHg, and alpha-stat management of pH was performed. Hypothermia was induced; the temperature at the bottom of the nasopharyngeal temprature was 28 °C. We used pulsatile perfusion assist to maintain cerebral circulation during CPB with IABP. A decrease in rSO_2_ was observed 162 min after the initiation of CPB. Our perfusionist increased the CPB pump flow from 2.2 L/min/m^2^ to 2.8 L/min/m^2^ in order to increase cerebral blood flow. Moreover, the concentration of sevoflurane was increased to 2 %. Yet, rSO_2_ desaturation (15 % reduction from baseline) was not improved. We decided to increase the depth-of-anesthesia with another dose of midazolam. After administration of 3 mg of midazolam, the rSO_2_ values increased from 67 to 73 % on the right side and from 71 to 74 % on the left side. During CPB, the lowest values (and variation) of rSO_2_ in the left and right forehead were 71 % (−2 %) and 67 % (−17 %), respectively. Mitral valve plasty was performed as planned.

At the weaning from the CPB, the disappearance of MR was confirmed by TEE; the weaning was not difficult. Pulmonary hypertension also improved (PAP was 26/12 mmHg, while systemic arterial pressure was 105/56 mmHg). After CPB, inhalation of sevoflurane (1–1.5 % of end-tidal concentration) was also continued. The rSO_2_ values were almost above 75 % on both sides and not below the awake rSO_2_ values (Fig. 2). CPB and aortic cross-clamping lasted 352 min and 289 min, respectively. On the completion of the surgery, the IABP was discontinued and sevoflurane administration was stopped. The patient was transferred to the intensive care unit with ventilator support under propofol sedation.

**Fig. 3:**
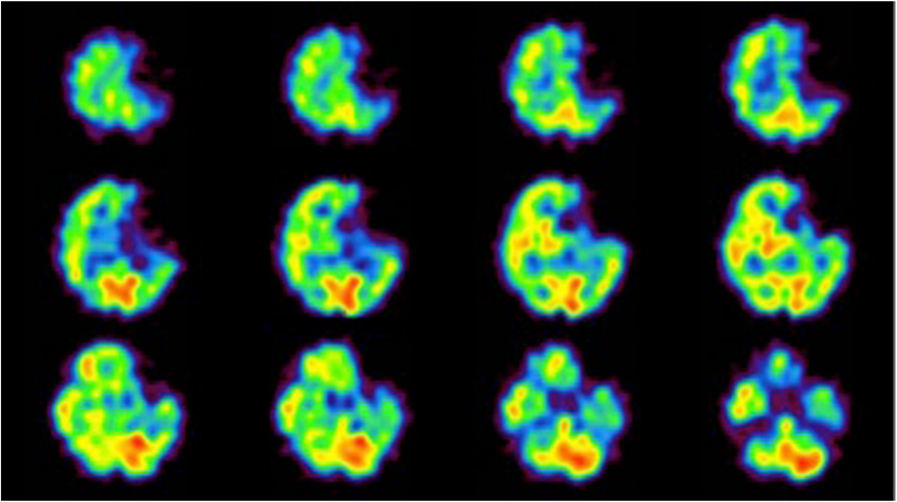
Postoperative single-photon emission computed tomography (SPECT) scan. The postoperative SPECT scan obtained from our hospital revealed that low cerebral blood flow lesions in the left cerebral hemisphere had not changed compared to the preoperative SPECT scan

On the 1st postoperative day (POD), the patient was weaned from the ventilator, and the patient did not complain about any new neurological deficits. We monitored the rSO_2_ of his forehead until the 2nd POD and no significant decrease (−20 %) of the rSO_2_ values was confirmed. The postoperative course was uneventful. On the 15th POD, single-photon emission computed tomography revealed that the low cerebral blood flow lesions had not changed (Fig. 3), and the patient was discharged from our hospital on the 16th POD.

## Discussion

We performed anesthesia for mitral valve plasty in a patient with cerebral infarction due to MMD using pulsatile perfusion of cardiopulmonary bypass with an assistance of IABP, without exacerbation of neurological complications.

Anesthetic management of a patient with MMD is rather challenging, and we have to keep several key-points in mind. Among them, maintaining normocapnea is most critical. Hypocapnia would induce brain ischemia and brain infarction, while hypercapnia would induce vasodilation and hyper-perfusion of the fragile vessels in the brain, which might cause brain hemorrhage. In patients with MMD, both vasoconstriction and vasodilation would not be preferable. That is why keeping normocapnea is quite important.

During CPB, PaCO_2_ was maintained between 45 and 50 mmHg with alpha-stat management of pH. This patient developed MMD not by the onset of cerebral hemorrhage but by the onset of cerebral infarction. We regarded that we should avoid hypocapnia or normocapnea, which would induce cerebral infarction, rather than hypercapnia, which would induce cerebral hemorrhage. But, we cannot ignore that hypercapnia would induce vasodilation and hyper-perfusion of the fragile vessels in the MMD brain, which might cause brain hemorrhage.

Usually, extracorporeal circulation is maintained by non-pulsatile perfusion, and non-pulsatile perfusion can induce ischemic injury, especially in organs under inadequate perfusion [5]. In patients with restricted cerebral arterial blood supply such as MMD, decreased cerebral perfusion pressure and non-pulsatile perfusion during CPB are risk factors for cerebral ischemia.

Kashima et al. [6] reported that high-pressure pulsatile perfusion assisted by IABP was effective for brain protection during coronary artery bypass grafting in a MMD patient. De Buysscher et al. [4] also reported that conversion from a non-pulsatile flow to a pulsatile flow resulted in a gradual increase in rSO_2_ values as opposed to a sudden decrease in rSO_2_ in an adult MMD patient undergoing CPB. In addition, Cheul-Hong et al. [3] reported up to 15 % fluctuations in rSO_2_ values during cardiac surgery in an MMD patient who was later discharged from the hospital without any complications. In pediatric patients undergoing CPB, pulsatile flow has advantages over nonpulsatile flow as measured by near-infrared spectroscopy and transcranial Doppler ultrasound, which may improve postoperative neurodevelopmental outcomes [7]. Owing to these previous reports, we selected the pulsatile flow method supported by IABP in our case and maintained the mean perfusion pressure above 70 mmHg.

The optimum anesthetic agent for the maintenance of anesthesia in MMD patients requiring cardiac surgery is a topic of much debate. Neither inhalational anesthetics (sevoflurane or isoflurane) nor intravenous anesthetic (propofol) presents strong clinical evidence for the efficacy of the outcome. Propofol suppresses cerebral metabolism and reduces cerebral blood flow [8]. In contrast, sevoflurane strongly dilates cerebral vessels and increases cerebral blood flow [9]. Moreover, several reports describe sevoflurane preconditioning against myocardial ischemia-reperfusion injury. Recently, it has been reported that sevoflurane has the potential preconditioning effect against cerebral ischemia [10], although obvious preconditioning effect of sevoflurane has not been proved clinically. In rats with transient cerebral ischemia, sevoflurane preconditioning protects mitochondria from cerebral ischemia-reperfusion injury and ameliorates long-term neurological deficits [11]. Thus, these properties may support sevoflurane use over propofol for intracranial steno-occlusive arterial disease, but choice of anesthetic in patients with MMD remains an open question.

A decrease in rSO_2_ was observed during the CPB. Currently, rSO_2_ is considered as the average of arterial oxygen saturation (SaO_2_) and internal jugular venous oxygen saturation (SjO_2_) in the measured region. Several reasons should be considered for changes of rSO_2_. It is also known to be influenced by the skin blood flow [12]. Cutaneous vasoconstriction induced by vasoconstrictor, such as phenylephrine and norepinephrine, or hypothermia during CPB possibly affects changes of rSO_2_.

In this case, we increased the pump flow and concentration of sevoflurane to increase cerebral blood flow, but these interventions were not effective. Secondly, we deepened the anesthesia level with a supplemental intravenous anesthetic. After another administration of midazolam, an improvement in bilateral rSO_2_ values was observed. We didn’t know why bilateral rSO_2_ was improved after administration of an intravenous anesthetic, but we cannot ignore the possibility that an intravenous anesthetic decreases the cerebral metabolic rate of oxygen consumption [7]. When regional cerebral oxygen desaturation continues during sevoflurane general anesthesia in patients with MMD, it might be efficacious to administer intravenous anesthetics concurrently. These management procedures limited the fluctuation of rSO_2_ values to within 17 % in our patient, which was nearly equal to that in a previous study [3], and prevented the development of cerebral deficits.

## Conclusion

Pulsatile flow supported by IABP during CPB had a possible beneficial effect for brain protection in patients with restricted cerebral arterial blood supply. Sevoflurane inhalation was continued during CPB with an expectation of its vasodilatory activity and its potential neuroprotective effect. We could maintain rSO_2_ at 80 % of baseline value during the surgical procedure, and we could not detect other neurological deficits. But, the further discussion is warranted whether inhalational anesthetic or intravenous anesthetic might be superior for intracranial steno-occlusive arterial disease. Thus, our case provides further evidence for the efficacious use of this anesthetic in MMD patients requiring cardiac surgery.

## Abbreviations

CPB, cardiopulmonary bypass; IABP, intra-aortic balloon pumping; MMD, moyamoya disease; MR, mitral regurgitation; PaCO_2_, arterial carbon dioxide partial pressure; PAP, pulmonary arterial pressure; POD, postoperative day; rSO_2_, regional cerebral oxygen saturation; TEE, transesophageal echocardiography
